# Development of a Speech Intelligibility Test for Children in Swiss German Dialects

**DOI:** 10.3390/audiolres16010016

**Published:** 2026-01-22

**Authors:** Christoph Schmid, Stefanie Blatter, Eberhard Seifert, Philipp Aebischer, Martin Kompis

**Affiliations:** 1 Department of Otorhinolaryngology, Head and Neck Surgery, Bern University Hospital, Inselspital, 3010 Bern, Switzerland; eberhard.seifert@insel.ch (E.S.); martin.kompis@insel.ch (M.K.); 2 Hearing Research Laboratory, ARTORG Center for Biomedical Engineering Research, University of Bern, Murtenstrasse 50, 3008 Bern, Switzerland; 3 Institut für Heilpädagogik, Pädagogische Hochschule Bern, Fabrikstrasse 8, 3012 Bern, Switzerland

**Keywords:** speech reception, native dialect, child, adaptive procedure, picture-pointing-task

## Abstract

Objective: This paper describes the development of a speech intelligibility test in Swiss German dialects, designed for children aged four to nine who are not yet familiar with standard German. Method: Suitable monosyllabic words and trochees in different Swiss German dialects were compiled, illustrated, and evaluated. Picture-pointing test procedures appropriate for children were developed. The selected test words and the pictures representing them were evaluated in a preliminary trial with forty-six normal-hearing children between two and nine years of age. Results: A set of 60 monosyllabic words and 40 trochees was recorded in four different Swiss German dialects as well as in standard German, resulting in a total of 500 recordings. Drawings were created to illustrate each word and found to be appropriate for children aged four years old or older. A non-adaptive and an adaptive test procedure using a weighted up–down method to measure speech reception thresholds in quiet and in noise were developed. Conclusions: A novel test to determine speech intelligibility in children in four different Swiss dialects was developed and evaluated in a pilot study. A validation study with more participants was designed to evaluate the test material and procedures.

## 1. Introduction

Speech intelligibility tests are important diagnostic tools when investigating hearing disorders. Tests for children and adults typically differ in terms of test material selection and test procedure. For children, all test items should be a part of their vocabulary. While adults are expected to repeat words they understand, children’s speech production lags behind speech comprehension [1]. Additionally, some children may be too shy to talk to strangers, especially if they are unsure whether they have understood a test item correctly.

Several tests in German are suitable for children [2,3,4,5,6]. These tests have been validated and are used clinically. However, in the German-speaking part of Switzerland, these speech intelligibility tests have limited application; parents usually speak a Swiss German dialect with their children who only learn standard German when they start school at the age of four. Even adults understand their native dialect better than foreign ones, but this effect is even more pronounced in children [7,8,9,10]. Although the capability to understand foreign accents starts developing in toddlers [11], it matures only in adolescence [12,13]. For Swiss children, adapting to standard German takes time because of the considerable differences between Swiss dialects and standard German [14,15]. Not only are words pronounced differently but their etymology can also differ, making some standard German words unfamiliar to young children. This is the main reason why standard German tests are of limited usefulness, as they often contain words that differ significantly from Swiss German. For example, the MATCH test [4] uses the words “Pferd” (horse) and “Butter” (butter), whereas the corresponding Swiss German words are “Ross” and “Anke”.

There are several different dialects in German-speaking Switzerland, which change gradually between regions and over time [16]. Consequently, we cannot count the dialects conclusively, but we can offer language tests in the dialects spoken around highly populated centers, such as Zurich or Basel, to which as many children as possible are reasonably familiar. We believe that the more familiar a child is with the dialect used in the test, the more accurate the results will be.

This report describes the development and design of a speech intelligibility test in quiet and noise for children in different Swiss German dialects.

## 2. Materials and Methods

The development of the speech test included several work packages, which depended on each other in various ways. Thus, the design process was often iterative and not as linear as the following division into separate tasks may suggest.

### 2.1. Design of Test Procedures

Our aim was to create a new test to measure speech intelligibility in quiet as well as in noise. A secondary goal was to provide examiners with at least one adaptive and one non-adaptive procedure. Furthermore, each test item should be represented by an unambiguous picture. Early on, we decided to design the test as a word test. To prevent fatigue, confusion, and lengthy response times, only a limited number of pictures should be presented at the same time for each presentation of a test item. Since the test is meant to be repeatable, it should be difficult to memorize the answers, even after several attempts. With these goals in mind, we compared, discussed, and optimized several designs.

### 2.2. Compilation of Test Items and Pictures

We compiled a set of test words, which had to meet all of the following criteria: (1) The word should be expected to be part of the vocabulary at least of four-year-olds, but preferably also of younger children. (2) It should be unambiguously representable with a picture. (3) The word must consist of either one or two syllables. (4) It must have the same number of syllables, the same meaning, and recognizably the same etymology in standard German and at least in the following Swiss dialects from Basel, Bern, Valais, and Zurich, but preferably in other Swiss dialects as well.

With these criteria in mind, a team consisting of a phoniatrician, a speech therapist, a pediatric audiologist, and an audiologist compiled a pool of 217 potentially suitable words. To this end, we did consult existing tests for children in German, but we also aimed to supplement the number of words with ones believed to be commonly known to young children. This original long list was subsequently thinned out in an iterative process consisting of discussions, drafting balanced lists, drawing, and, if necessary, redrawing pictorial representations and evaluating the test items and pictures with children. The criteria to reject words from the long list were as follows (in this order): (1) different etymology or different number of syllables in different dialects, (2) cannot be reasonably expected to be in the vocabulary of the majority of four-year-olds, and (3) difficulties to find an unambiguous pictorial representation.

### 2.3. Verification of the Suitability of the Test Items and Pictures

In a pilot study, the suitability of the test words and their pictorial representations in the final pool was evaluated. The test words were presented live, orally, and clearly, with lipreading allowed to 46 normal-hearing children (18 girls and 28 boys) aged two to nine years (mean 4.6 years, SD 2.0 years). The procedure and response options are described in Section 3.4. All study participants as well as their parents gave informed consent to participate in this study.

### 2.4. Recording, Equalization and Generation of the Noise Signal

The test words were recorded in a professional studio by five female native speakers in four Swiss dialects (Basel, Bern, Valais, Zurich) and in standard German. As an example, the verb “to swim”, is pronounced and recorded as [ʃvɪmən] in standard German, [ʃvʏmːə] in the Bernese, [ʃvʏmə] in the Zurich, and [ʃvɪmːʊ] in the Valais dialect. The female speakers were young professionals working as either radio presenters or actresses, and they pronounced the words in a normal tone and at a normal pace. In addition to the test words, the phrase “show me” was recorded in each dialect. A sampling rate of 44.1 kS/s and a dynamic range of 32 bits were used. We then measured the A-weighted impulse max-hold level for each word (Audioanalyzer software dBFA 4.8, 01 dB-metravib, Lyon, France) and equalized the levels of all test words (Matlab 2022b, the Mathworks, Natick, MA, USA). Calibration in decibel sound-pressure-level (dB_SPL_) was performed using RadioEar DD450 headphones, a B&K 4153 artificial ear with a B&K type 4180 microphone (Brüel & Kjær, Nærum, Denmark), a Norsonic type 1207 preamplifier, and a Norsonic Nor116 sound level meter (Norsonic AS, Tranby, Norway).

To allow speech tests in noise, a noise signal was generated for each dialect. All test words from each recorded dialect were superimposed 100-fold to form speech spectrum-shaped noises, the levels of which were adjusted to the words (A-weighted, LEQ slow). Recording and post-processing were carried out in accordance with [17].

## 3. Results

### 3.1. Test Material

The original pool of test words was pruned down to a total of 100 test words, which were organized into 10 lists of 10 words each. Four of the lists consist of monosyllabic words and six consist of trochees. It was not possible to fully balance the lists phonetically, primary due to the differences between the dialects. However, within each list of each group (i.e., monosyllabic words or trochees), the distribution of the initial phonemes, the number of sibilants, and the vowels are either identical or distributed as evenly as possible. Furthermore, each list contains an equal number of words from the following categories: animals, body parts or persons, food, verbs (trochees only), and nouns. A drawing was created for each of the 100 test items.

### 3.2. Suitability of the Test Items and Pictures

**Figure 1:**
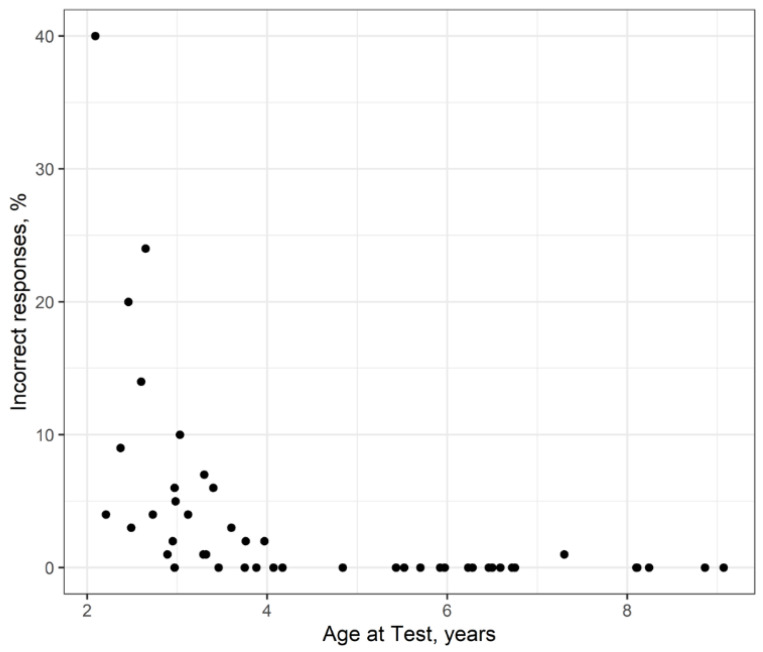
Percentage of incorrect responses (live oral presentation, response by picture pointing).

Figure 1 shows the percentage of incorrectly identified pictures by the 46 normal-hearing children of the pilot test, when using all 100 words and their pictorial representations of the final selection. At this stage, the words were spoken live and clearly. Pictures were presented in groups of six, using the panels described in Section 3.4. The tests took place either at the children’s homes or at our hospital’s audiology department. One examiner led the test, while another recorded the answers. At least one of the child’s parents was always present. Every effort was made to ensure the children felt comfortable throughout the testing process.

Generally, the test words were well-known, and the corresponding pictures were easy to point out for the children aged four and above. Only one word was not identified correctly by one child in this age group. Under the age of four, more errors occurred, rather evenly distributed over the different test items. Nevertheless, even children as young as three years recognized 90% or more of the words presented. The three test items missed most frequently were “creek” (five times), “nest” (four times) and “cup” (four times as well). The test seemed to be easy to perform and even fun for the children.

### 3.3. Recordings of Test Items

**Figure 2:**
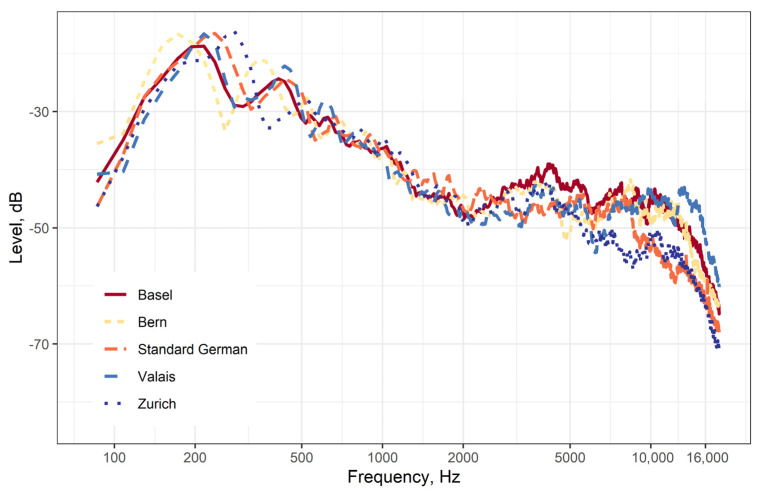
Long-term frequency spectrum of all the test words for the different recordings.

As shown in Figure 2, the long-term frequency spectra of all test words differ somewhat between the five recorded dialects, mainly above 2000 Hz.

There were also differences in the average duration of the test words. Table 1 presents a synopsis of the mean word lengths of all 100 test words for each version of the test. The mean lengths are similar for most dialects, except for Bern, which is higher.

**Figure 3:**
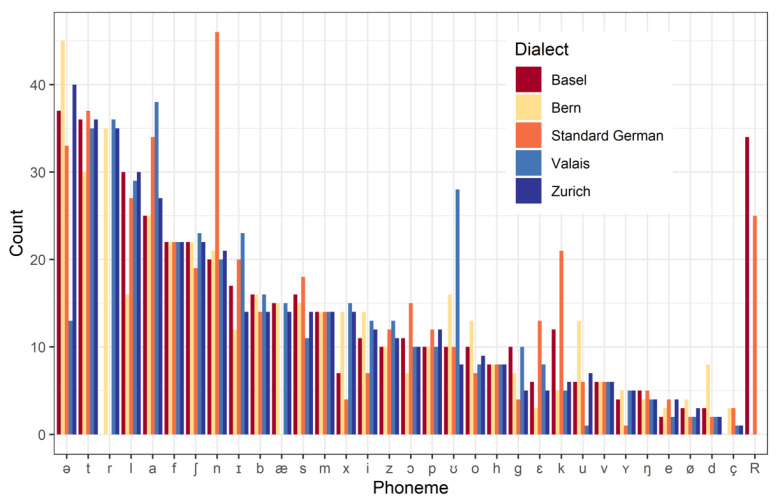
Phoneme distribution of the test words for different dialects.

Considerable differences between the dialects can be seen in the phoneme distribution, shown in Figure 3. For this analysis, each test word was transcribed using the International Phonetic Alphabet. As can be seen, there are significant differences in some phonemes, such as [r], [n], and [R]. These differences are genuine and cannot easily be balanced across the different versions.

### 3.4. Test Procedures

The recorded material, along with the pictures, can be used in various ways. We have designed both non-adaptive and adaptive test procedures.

**Figure 4:**
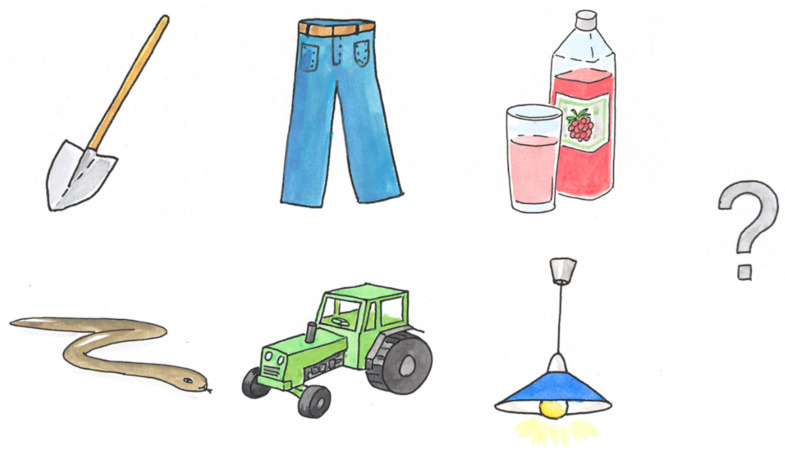
Example of a response screen for trochees with the option “don’t know” to the right.

All tests are designed as picture-pointing tasks in which each test word is preceded by the announcement “show me”, which is always 12 dB louder than the signal. Simultaneously, six pictures are shown to the child on a touch-sensitive screen where the answer can be selected. The response panels were compiled in such a way that concepts or items which might be confused, such as wolf and dog, or lake and stream, were avoided in the same panels. In a strict forced-choice selection with six pictures, some children might find it challenging to guess if they did not understand the word. Several researchers do not recommend encouraging children to guess [18,19]. For this reason, as a seventh alternative the option “don’t know” is presented on the screen as a question mark. Figure 4 shows an example of a response panel.

A software package was developed to allow an efficient presentation of the words, to save the results in a database, and to calculate the results.

#### 3.4.1. Non-Adaptive Testing

In non-adaptive testing, each 10-word list is presented at a fixed level, and the number of correctly recognized words is counted. The speech reception threshold (SRT) in quiet can be estimated by interpolating the two results adjacent to the level of 50% understanding. A similar procedure can be used in noise.

**Figure 5:**
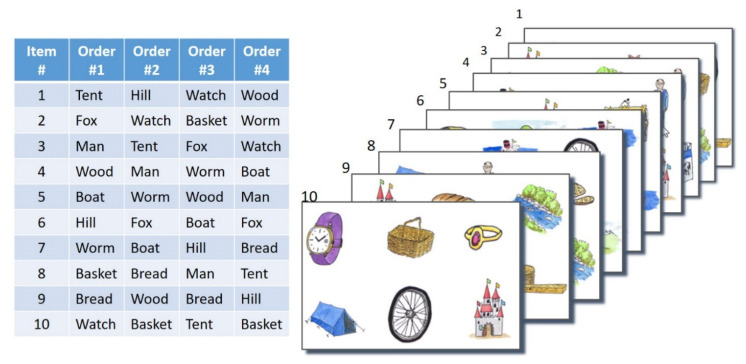
Principle of the non-adaptive procedure, example of one list: the test can be performed in four different orders, using the same 10 response screens—one for each item (the words have been translated into English and are all monosyllabic in German).

We have defined four different sequences or word orders within each of the groups of 10 test words. Furthermore, we have created 10 response panels, each containing six pictures, for each test list. Figure 5 shows the compiled material for one list schematically. The 10 response panels are presented successively along with each new test word. These panels are designed to be used in the same order with any of the four word orders. Thus, even for the same response screen, different answers may be correct. Additionally, the placement of the six pictures varies randomly with each presentation, making it difficult to predict the correct answer based solely on the pictures seen on a response screen.

#### 3.4.2. Adaptive Testing

The same test material can be used for adaptive test procedures, in which the presentation level of each new test item depends on the previous responses. This approach allows for the estimation of both the SRT and the entire psychometric function. Similarly, an adaptive test procedure can be used to determine the SRT in noise by varying the signal-to-noise ratios (SNRs) of the test items instead of their presentation levels.

**Figure 6:**
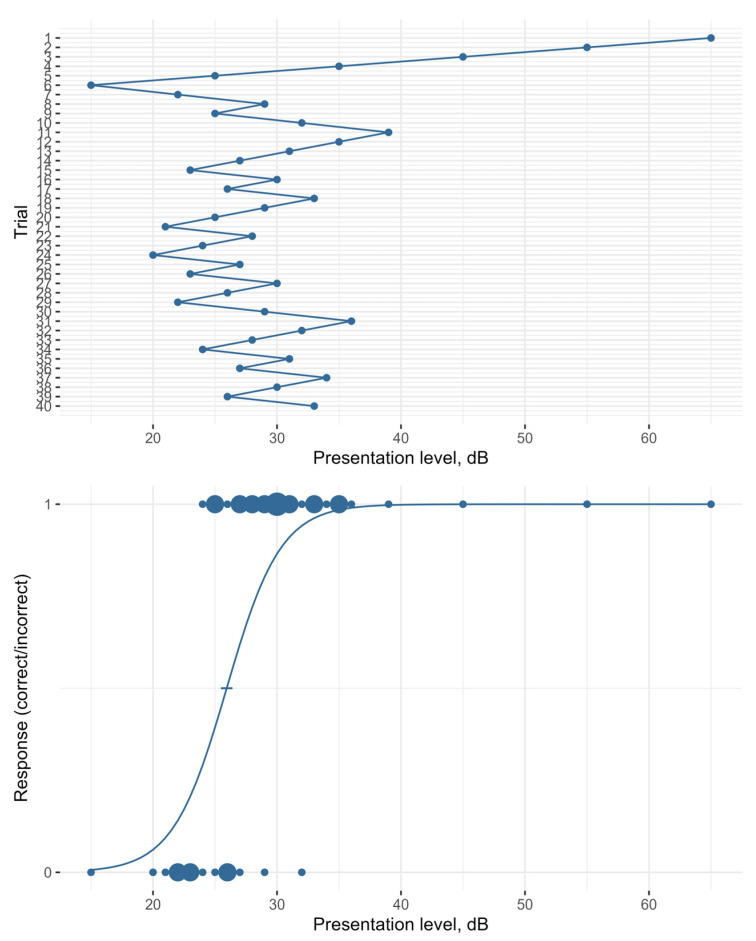
Example of a test-track with 40 trials (**upper panel**) and the obtained responses with the corresponding psychometric function (**lower panel**). The number of responses is represented by the symbol size.

We propose a weighted up–down procedure [20]. For tests in quiet, the presentation level of the next test item decreases by N dB if the last word was correctly understood and increases by M dB if it was not. Similarly, for tests in noise the SNR increases or decreases by given step sizes. Figure 6 shows the principle in the upper panel. To allow for more items per test run, several lists of either monosyllabic words or trochees can be combined.

The optimal step sizes, N and M, depend on two factors: (1) the targeted percentage of words understood in the steady state and (2) the slope of the discrimination curve. To maintain high motivation, we aim for 60 to 75% correct answers rather than the 50% often used for adults. Through computer simulations, we found that smaller step sizes are more suitable for steeper slopes of the discrimination curve, while larger step sizes are more suitable for shallower slopes. For our test material, the slopes in quiet and in noise are not yet known. We simulated tests with different combinations of step sizes. A reasonable point to start with might be N = 4 dB and M = 7 dB in quiet and N = 2 dB and M = 5 dB in noise. These settings lead to an average correct answer rate of 64% in quiet and 71% in noise.

According to the following equation

(1) $$p=\frac{1}{1+e^{-4\beta (L-\alpha )}},$$

The SRT and the psychometric function can be estimated by fitting a logistic function to the responses using a generalized linear model (GLM) with *p* = probability of correct response at stimulus level *L*, *α* = threshold, and *β* = slope.

Figure 6 gives an example of responses and the corresponding logistic function in the lower panel. The GLM not only allows to extract the SRT and the slope from its coefficients but can also indicate the reliability of the fit by its confidence interval or standard error (*SE*). The *SE* for any linear combination of the model coefficients can be determined by matrix multiplication. The SRT’s *SE* is calculated according to the following equation

(2) $$SE=\sqrt{\left[\begin{array}{cc} 1 & SRT \end{array}\right]\times cov\left[X\right]\times \left[\begin{array}{c} 1\\ SRT \end{array}\right]}$$

where *X* is the coefficient matrix and *SRT* the speech reception threshold level [21].

## 4. Discussion

Swiss German dialects differ significantly from standard German in pronunciation, phonetics, and vocabulary, as illustrated, e.g., by Figure 3. We believe that testing in native dialects may be important for the accurate assessment of a child’s speech reception.

In this work, we present newly developed test material—recorded test items and their pictorial representation—for the testing of speech intelligibility in Swiss German-speaking children. To our knowledge, this is the first such test in Swiss German. Switzerland is a small country, but the same underlying principles might be useful in other regions of the world where different dialects are used.

The long-term spectra of the recorded dialects show some variation but are generally comparable to those published for standard German [22]. To our knowledge, there are no data on Swiss dialects for comparison. Similarly, we are not aware of any such data for Swiss dialects to compare the phoneme distribution of the test words. Phoneme distributions for standard German were determined by Meier [23] and Hug [24], but this data will most probably not match the Swiss dialects.

A comparison of the different dialects within our test material reveals some differences in terms of phoneme distribution. For example, the Basel dialect is the only recorded Swiss dialect that expresses the phoneme [R], which is quite frequent in standard German but appears as [r] in most other Swiss dialects. Notably, 15% of the words are verbs in their basic form, which typically possess identical endings that vary distinctly between dialects. These verb endings peak in the phoneme diagram as [n] for standard German and as [ʊ] for the Wallis dialect while most verbs in the other dialects end with [ə].

The familiarity and the neighborhood density of words influence their intelligibility [25,26]. Both effects are minimized in the presented test. Familiarity is achieved through deliberate word selection, and the limited response options exclude possible neighbors. One alternative approach would have been to use minimal pairs. However, this approach is difficult when several dialects are involved.

Our aim was to be able to test young children. The results of the pilot study suggest that the test words were familiar to normal-hearing children ages four and up and that they could easily identify the corresponding pictorial representations.

We propose non-adaptive and adaptive test procedures to administer the material. Adaptive procedures tend to be faster than repeated measurements at fixed levels and allow for more correct answers, thus keeping the child’s motivation high. Additionally, they may provide an indicator of the reliability of the result. It remains to be seen which procedure will be used more frequently or be more useful in clinical practice. Since the steepness of the discrimination curves is not yet known, the suggested step sizes may not be optimal.

### 4.1. Limitations

We are aware of several limitations. So far, we have recordings in four dialects and standard German. While these dialects cover a considerable part of Switzerland, some regions will have to use the next closest dialect if no more recordings are made. Also, we currently do not know how similar the test results are in different dialects.

Most importantly, an evaluation of normative curves and test comparability across different dialects is required. To this end, a multicenter evaluation study has been designed. The study will include 180 children aged four to six and is registered as NCT06567613 on ClinicalTrials.gov. We hope to present the results within a year. This study may help address another limitation of our test material. So far, the ten lists have been equalized in terms of loudness levels, distribution of initial phonemes, number of sibilants and vowels, and distribution of word categories. However, we do not yet know how similar they are in terms of intelligibility.

### 4.2. Summary

We presented new test material consisting of recordings of test words in four dialects and standard German, along with accompanying pictorial representations suitable for children aged four years and older. We have designed adaptive and non-adaptive test procedures. A multicenter study to evaluate the test was designed and registered.

## Figures and Tables

**Table 1:** Mean word length in seconds for recordings in four Swiss dialects and standard German.

	Basel	Bern	Standard German	Valais	Zurich
Mean word length	0.73 s	0.87 s	0.73 s	0.71 s	0.70 s

## Data Availability

The data, recordings, and drawings from this study will be made available by the corresponding author upon reasonable request. He can also provide the software package to present the recordings and drawings, control the adaptive procedure, and calculate the results.

## Abbreviations

The following abbreviations are used in this manuscript:

dB_SPL_	decibel sound-pressure-level
SNR	signal-to-noise ratio
SRT	speech reception threshold
GLM	generalized linear model
SE	standard error
